# A Unique Case of Inferior Vena Cava Aneurysm Complicated with Pulmonary Embolism and Cerebral Infarction

**DOI:** 10.3390/jcdd8110147

**Published:** 2021-11-04

**Authors:** Hyunglan Chang, Jinkun Bae, Tae Nyoung Chung

**Affiliations:** Department of Emergency Medicine, CHA Bundang Medical Center, CHA University School of Medicine, Seongnam 13496, Korea; senna7@cha.ac.kr (H.C.); galen97@chamc.co.kr (J.B.)

**Keywords:** inferior vena cava, venous aneurysm, cerebral infarction, pulmonary embolism, paradoxical embolism

## Abstract

Inferior vena cava (IVC) aneurysms rarely occur. They are commonly detected incidentally since they present with mild or no symptoms. This was the first study to report a fatal case of a saccular IVC aneurysm with pulmonary embolism and cerebral infarction. The patient developed cardiac arrest five minutes after arriving at the emergency department, and spontaneous circulation was restored after two minutes of cardiopulmonary resuscitation. Computed tomography scans of the brain, chest, and abdomen–pelvis were obtained. The patient was diagnosed with a saccular aneurysm of the IVC measuring 8 × 11 cm, massive embolism of both pulmonary arteries, and cerebral infarction. An electroencephalogram, taken on the third day of hospitalization, suggested brain death, and the patient died on the eleventh day of hospitalization. This case report highlights that an IVC aneurysm with pulmonary embolism can be associated with paradoxical emboli-induced cerebral infarction, which is fatal.

## 1. Introduction

Venous aneurysms are uncommon. In particular, inferior vena cava (IVC) aneurysms are rare, with approximately 70 reported cases [1,2]. Most IVC aneurysms are incidentally identified among asymptomatic or mildly symptomatic patients, the most common symptoms are abdominal pain and symptoms related to deep vein thrombosis [3]. However, some cases, which occurred together with pulmonary embolism, were reportedly fatal [4]. We report a unique and fatal case of a saccular IVC aneurysm with pulmonary embolism and cerebral infarction in a 58-year-old woman.

## 2. Case Presentation

**Figure 1:**
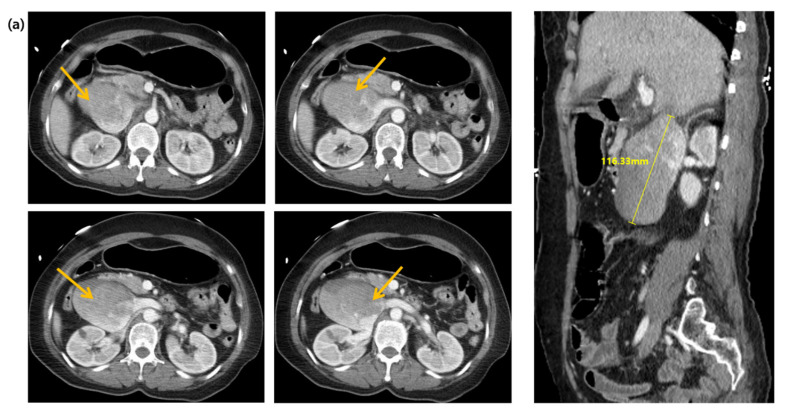

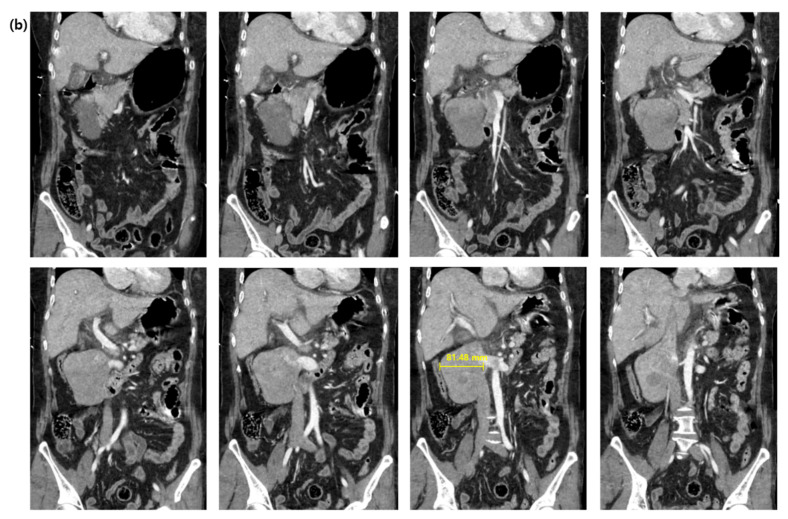
Computed tomography images of the abdomen and pelvis in (**a**) the axial and sagittal planes, and (**b**) the coronal plane. Arrows indicate an inferior vena cava aneurysm.

**Figure 2:**
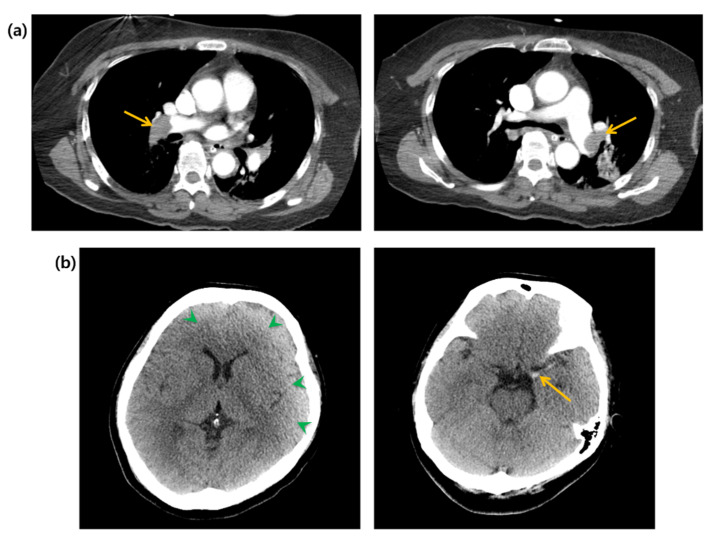
(**a**) Computed tomography of the chest. Arrows indicate massive pulmonary embolisms. (**b**) Computed tomography of the brain. Green arrowheads indicate widely decreased gray matter density. Yellow arrow indicates hyperdense middle cerebral artery sign.

A 58-year-old woman with an unremarkable medical history visited the emergency department (ED) for worsening dizziness and dyspnea, noted one day before. The patient was alert and had normal peripheral oxygen saturation (SpO2) when the paramedics arrived. However, her mental state and oxygen saturation level declined during transport to the hospital. She had an SpO2 of 83% despite receiving 100% oxygen supplementation through a face mask with a reserve bag. The blood pressure was not measured due to a weak pulse. The patient was drowsy upon arrival at the ED. Cardiac arrest with pulseless electrical activity occurred after five minutes, and spontaneous circulation was restored after two minutes of cardiopulmonary resuscitation. A computed tomography (CT) of the brain, chest, and abdomen–pelvis (AP) was taken two hours after arrival. The AP-CT revealed an 8 × 11 cm saccular aneurysm involving the left and right renal veins at the infrahepatic level of the IVC (Figure 1). The chest CT revealed massive pulmonary embolisms involving both distal main pulmonary arteries (Figure 2a). A hyperdense middle cerebral artery (MCA) sign indicating thrombosis of the M1 MCA segment and widely decreased gray matter densities in the left hemisphere and right frontal lobe were detected on the brain CT (Figure 2b) [5]. A tissue plasminogen activator was administered two and a half hours after arrival, and the patient was admitted to the intensive care unit. The patient developed a seizure; therefore, a follow-up brain CT was performed after six hours. This showed a broad infarcted area involving most of the cerebrum, except for the thalamus and right occipital lobe. An electroencephalogram taken two days after admission suggested the possibility of brain death, and a bedside transthoracic echocardiography performed on the fourth day of hospitalization showed an enlarged RV with reduced RV systolic function. The patient’s family decided against a further evaluation or advanced treatment; therefore, the patient was conservatively managed at this point. Normal blood pressure was not achieved despite administering high-dose inotropic agents during the seventh day of hospitalization, and the patient died on the eleventh day of hospitalization.

## 3. Discussion

This was the first case report of a fatal IVC aneurysm, occurring in combination with pulmonary embolism and cerebral infarction.

An IVC aneurysm is defined as more than twice the normal IVC diameter, and its etiology is unclear [6,7]. Several studies have suggested trauma, inflammation, and congenital problems as possible causes [8,9,10,11,12]. Gradman and Steinberg proposed a classification system for IVC aneurysms based on their anatomic location and the presence of venous obstruction as follows [13]: type I, which refers to suprahepatic aneurysms without a venous obstruction; type II, which includes aneurysms associated with a venous obstruction above or below the hepatic veins; type III, which comprises infrarenal aneurysms without a venous obstruction; type IV, which consists of aneurysms with miscellaneous characteristics. Montero-Baker et al. suggested a management guideline for IVC aneurysms based on this classification system [1]. The patient, in this case, had a type III IVC aneurysm, which requires active interventions, such as surgical resection, ligation, and endovascular management, because type II-IV aneurysms have flow dynamics that cause venous stasis resulting in thrombosis. In this case, fatal thrombotic complications had already occurred at the time of arrival, and the patient died before the indicated interventions could be considered.

Cerebral infarction rarely coexists with pulmonary embolism; in contrast, deep vein thrombosis occurs as a complication of cerebral infarction, resulting in pulmonary embolism [14]. Moreover, cerebral infarction cannot be directly caused by an IVC aneurysm. However, paradoxical embolism, the arterial occlusion caused by venous thrombosis in patients with a right-to-left shunt, accounted for the occurrence of pulmonary embolism and cerebral infarction in this case of IVC aneurysm. Paradoxical embolism is suspected if arterial embolism and venous thromboembolism coexist, and this was frequently observed among patients with a stroke of unknown etiology [15,16]. Most cases of paradoxical embolism are associated with a patent foramen ovale (PFO). It is more prevalent among cerebral ischemia patients than in the general population [17]. Based on this finding, although a PFO screening was likely indicated for our patient, it could not be performed because she became brain-dead before a further evaluation. A previous study suggested that the earlier detection of an atrioventricular shunt might be helpful if transesophageal echocardiography is performed during initial resuscitation [18].

In this case, an IVC aneurysm could be a bystander finding, which coexisted with cerebral infarction caused by paradoxical emboli from another venous thrombosis. However, we could not find any other suspicious cause of thromboembolism in the imaging studies or previous medical history of the patient, except for large thrombi inside the IVC aneurysm. Hence, the most appropriate putative mechanism underlying cerebral infarction, in this case, is paradoxical embolism caused by thrombi inside the IVC aneurysm. Most importantly, this is the first case report of a comorbidity of an IVC aneurysm and cerebral infarction regardless of causal relation.

In conclusion, this case highlights that IVC aneurysms can result in the comorbidity of pulmonary embolism and cerebral infarction, both of which are fatal. Hence, IVC aneurysms should be managed carefully to avoid fatal complications, such as pulmonary and paradoxical embolisms. In addition, a paradoxical embolism and IVC aneurysm should be considered in patients presenting with symptoms of cerebral infarction and unexplained hypoxemia.

## Data Availability

Data are contained within the article.

## References

[1] Montero-Baker M.F., Branco B.C., Leon L.L., Labropoulos N., Echeverria A., Mills J.L. (2015). Management of inferior vena cava aneurysm. J. Cardiovasc. Surg..

[2] Wang M., Wang H., Liao B., Peng G., Chang G. (2021). Treatment strategies for inferior vena cava aneurysms. J. Vasc. Surg. Venous Lymphat. Disord..

[3] Woo K., Cook P., Saeed M., Dilley R. (2009). Inferior Vena Cava Aneurysm. Vascular.

[4] Van Ieperen L., Rose A. (1990). Idiopathic aneurysm of the inferior vena cava. A case report. S. Afr. Med. J..

[5] Mullins M.E. (2005). The Hyperdense Cerebral Artery Sign on Head CT Scan. Semin. Ultrasound CT MRI.

[6] McDevitt D.T., Lohr J.M., Martin K.D., Welling R.E., Sampson M.G. (1993). Bilateral Popliteal Vein Aneurysms. Ann. Vasc. Surg..

[7] Moustafa S.E., Mookadam M., Khandheria B.K., Mookadam F. (2007). Inferior Vena Cava Aneurysm. J. Am. Soc. Echocardiogr..

[8] Elliot A., Henn A., Pamuklar E., Rivero H., Hyslop W.B., Semelka R.C., Burke C.T. (2006). Aneurysm of the inferior vena cava: Case report. Abdom. Imaging.

[9] Furukawa T., Yamada T., Mori Y., Shibakiri I., Fukakusa S., Jitsukawa K., Ihori M., Tamaki M. (1986). Idiopathic aneurysm of inferior vena cava: CT demonstration. J. Comput. Assist. Tomogr..

[10] Koc Z., Oguzkurt L. (2007). Interruption or congenital stenosis of the inferior vena cava: Prevalence, imaging, and clinical findings. Eur. J. Radiol..

[11] Moncada R., Demos T.C., Marsan R., Churchill R.J., Reynes C., Love L. (1985). CT Diagnosis of Idiopathic Aneurysms of the Thoracic Systemic Veins. J. Comput. Assist. Tomogr..

[12] Sullivan V., Voris T., Borlaza G., Lampman R., Sood M., Shanley C. (2002). Incidental Discovery of an Inferior Vena Cava Aneurysm. Ann. Vasc. Surg..

[13] Gradman W.S., Steinberg F. (1993). Aneurysm of the Inferior Vena Cava: Case Report and Review of the Literature. Ann. Vasc. Surg..

[14] Fujino Y., Kawasaki T., Kawamata H., Tamura A., Shiga K., Oyamada H. (2020). Cerebral Infarction with Pulmonary Thromboembolism Due to Immobilization. Intern. Med..

[15] Itoh T., Matsumoto M., Handa N., Maeda H., Hougaku H., Tsukamoto Y., Kondo H., Tanouchi J., Kamada T. (1994). Paradoxical embolism as a cause of ischemic stroke of uncertain etiology. A transcranial Doppler sonographic study. Stroke.

[16] Travis J.A., Fuller S.B., Ligush J., Plonk G.W., Geary R.L., Hansen K.J. (2001). Diagnosis and treatment of paradoxical embolus. J. Vasc. Surg..

[17] Koutroulou I., Tsivgoulis G., Tsalikakis D., Karacostas D., Grigoriadis N., Karapanayiotides T. (2020). Epidemiology of Patent Foramen Ovale in General Population and in Stroke Patients: A Narrative Review. Front. Neurol..

[18] Vetrugno L., Pompei L., Zearo E., Della Rocca G. (2014). Could transesophageal echocardiography be useful in selected cases during liver surgery resection?. J. Ultrasound.

